# The emerging role of complex modifications of tRNA^Lys^_UUU_ in signaling pathways

**DOI:** 10.15698/mic2015.01.185

**Published:** 2015-01-05

**Authors:** Patrick C. Thiaville, Valérie Crécy-Lagard

**Affiliations:** 1 Genetics and Genomics Graduate Program, University of Florida, Gainesville, Florida, USA.; 2 University of Florida Genetics Institute, Gainesville, Florida, USA.; 3 Institut de Génétique et Microbiologie, Université of Paris-Sud, Orsay, France.; 4 Department of Microbiology and Cell Science, University of Florida, Gainesville, Florida, USA.

**Keywords:** tRNA, GCN4, thiolation, UPR, TOR

## Abstract

Coordination of cell growth with nutrient availability, in particular amino acids, is a central problem that has been solved by the implementation of complex regulatory cascades. Although the specific regulatory mechanisms differ between kingdoms and species, a common theme is the use of tRNA molecules as sensors and transducers of amino acid starvation. In many bacteria, amino acid starvation leads to high levels of uncharged tRNAs, a signal for the synthesis of the stringent response’s alarmones, halting transcription of stable RNAs and inducing the synthesis of amino acid synthesis pathways 1. In gram-positive Bacteria (as well as the Deinococcus-Thermus clade), uncharged tRNAs bind structures (T-boxes) in the leader sequences of mRNA encoding gene, activating the expression of genes involved in amino acid metabolism 2. In eukaryotes, the conserved General Amino Acid Control (GAAC) response is triggered by shortage of amino acids that leads to the binding of uncharged tRNAs to Gcn2 kinase and, through a cascade of events, to the activation of the central activator of amino acid synthesis genes, Gcn4 3. As the study by Scheidt *et al. *4 and several other recent studies in this field reveal, variations in charging levels are not the only mechanism by which tRNAs play a role in amino acid starvation responses; levels of post-transcriptional modifications also seem to play major roles.

The anticodon-stem-loop (ASL) of tRNAs drives decoding by interacting directly with the mRNA codon (Fig. 1). Modifications of the ASL are the most distinct and chemically complex of all RNA modifications 5. They are required for accurate codon recognition and translocation, enhance aminoacylation properties of tRNAs, and prevent ribosomal frameshifting 5. In eukaryotes, two of the most complex ASL modifications are N6-threonylcarbamoyladenosine (t^6^A) and 5-methoxycarbonylmethyl-2-thiouridine (mcm^5^s^2^U). The first modification is found at position 37 of most tRNAs decoding ANN codons, just before the residue that interacts with the first base of the codon. The second modification is found at position 34, the residue that decodes the 3^rd^ base of the codon, of tRNA^Lys^_UUU_, tRNA^Glu^_UUC_, and tRNA^Gln^_UUG_ (Fig. 1). Both these modifications are found in tRNA^Lys^_UUU_ and affect translation efficiency 5. In addition, several other striking parallels can be made between the two.

t^6^A and mcm^5^s^2^U are both synthesized by elaborate multimeric complexes that were first thought to be transcription factors. The KEOPS complex (Kinase, putative Endopeptidase and Other Proteins of Small size) in combination with Sua5 is involved in the synthesis of t^6^A 6, and the Elongator (ELP) complex in combination with the URM1 pathway enzymes (Uba4, Urm1, Ncs2/Ncs6) is involved in the synthesis of mcm^5^s^2^U 5. *Saccharomyces cerevisiae* mutants lacking t^6^A or mcm^5^s^2^U display many similar phenotypes. For example, telomere shortening is observed in the absence of either modification 7,8. In the case of ELP, it is now firmly established that most of the ELP phenotypes are due to the absence of the modified base, as the phenotypes are suppressed by overexpressing the tRNA targets 7,8,9. For example, the levels of the Sir4, a regulator involved in telomere maintenance and enriched in AAA (Lys) codons, are decreased in mcm^5^s^2^U deficient strains. Overexpression of tRNA^Lys^_UUU_ suppresses the telomere shortening phenotype 8. In *Schizosaccharomyces pombe,* key regulators, including the two subunits Atf1 and Pcr1 of the central regulator of the core environmental stress response 10 and the cell division regulator Cdr2 11, have an over-representation of AAA codons over other Lys codons, and their efficient translation is dependent on mcm^5^s^2^U levels. More generally, proteins involved in translation initiation and elongation are enriched in AAA codons, and reduced in cells lacking mcm^5^s^2^U 12,13. It is not yet known if the levels of these proteins enriched in AAA codons also require t^6^A for efficient translation in yeast, but it has been shown in mammals that a decrease in sulfur modified form of t^6^A (ms^2^t^6^A) on tRNA^Lys^_UUU _leads to lower levels of proinsulin 14.

**Figure 1:**
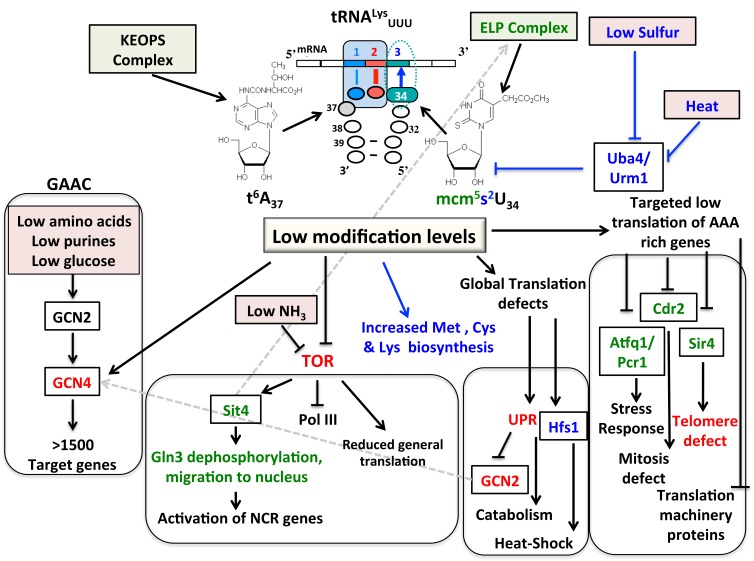
FIGURE 1: Possible cascade of events triggered when s^2^U, mcm^5^U or t^6^A levels are reduced in tRNA^Lys^_UUU_. Alteration in the levels of mcm^5^s^2^U occurring at position 34 of the tRNA (wobble position) and t^6^A occurring at position 37 (adjacent to the first base of the anti-codon) have global cellular affects. Reduction in either modification alters many regulatory cascades with the text color indicating the modification responsible for the change (red indicates expression change seen in t^6^A and mcm^5^s^2^U mutants). Solid lines represent experimental data; dashed lines indicated potential points of regulation

Another phenotype shared by a t^6^A or a mcm^5^s^2^U deficient *S. cerevisiae* derivatives is the *GCN2 *independent activation of *GCN4 *15,16. As the activation of the GAAC leads to a major reprograming of transcription (>500 genes are induced and >1000 are repressed) 17, this makes it difficult to interpret whether specific phenotypes observed in these tRNA modification mutants are due to direct or indirect effects. This is made all the more problematic given that another central regulator, the Target of Rapamycin (or TOR), is also affected by both t^6^A and mcm^5^s^2^U.

The TOR kinases regulate the balance between protein synthesis and protein degradation in response to nutrient quality and TOR activity is inhibited by low nitrogen conditions, caffeine or rapamycin 18. In yeast, reduced TOR levels increase levels of the Sit4 phosphatase, which dephosphorylates TOR targets such as the regulator Gln3. Unphosporylated Gln3 will then relocate to the nucleus to activate genes required for growth on low quality nitrogen sources. The following links between TOR and mcm^5^s^2^U have previously been made: 1) *ELP* mutants are sensitive to rapamycin and caffeine 16; 2) deletion of *SIT4* leads to rapamycin hypersensitivity and resistance to zymocin (a tRNase that recognizes mcm^5^s^2^U and cleaves tRNA^Lys^_UUU_ leading to cell death) 19 because Sit4 activates the ELP complex by phosphorylation 20. As shown by Scheidt *et al.* 4, the Elongator mutants mislocate Gln3 leading to rapamycin hypersensitivity, a phenotype that can be suppressed by over-expression of the tRNAs modified by mcm^5^s^2^U, suggesting that proper tRNA modification affects Gln3 localization and subsequent activation of the nitrogen catabolite repression (NCR) response. Mislocalization of Gln3 occurs both in *elp3*∆ (removing the mcm^5^ moiety) and in *urm*∆ (s^2^ moiety). A link between t^6^A synthesis enzymes and TOR has also recently been seen in flies. The Glavic group showed that lowering levels of one of the subunits of the KEOPS complex (Bud32) reduces TOR phosphorylation of S6K (*SCH9* in yeast) required for TOR dependent regulation of ribosome biogenesis 21.

How Gcn4 and TOR signaling depend on t^6^A and mcm^5^s^2^U is still far from understood at the molecular level. Are the Gcn4 activation and TOR repression in strains lacking these modifications due to direct effects caused by poor translation of specific proteins or are they part of general stress responses caused by translation inaccuracy and protein misfolding? The reality might lie in a combination of responses as in addition to the targeted effects described above, low mcm^5^s^2^U increases levels of proteins involved in proteasomal degradation 12. In addition, s^2 ^synthesis proteins in *S. cerevisiae* are unstable at high temperature and reduced levels of the modification lead to activation of the heat-shock response regulator (Hsf1) through the synthesis of unfolded proteins (Fig. 1) 22. Finally, silencing both t^6^A synthesis genes Bud32 and Kae1 in flies activates the Unfolded Protein Response (UPR) 23 and mutations of the thiolation enzyme leading to the formation of ms^2^i^6^A in mouse led to an increase of the Endoplasmic Reticulum (ER) stress response 14.

Because the synthesis of the t^6^A and mcm^5^s^2^U modifications of tRNA^Lys^_UUU_ draw on primary metabolism intermediates 24, it is tempting to propose that these could serve as sensing signals linking metabolism and translation. One recent example of such an integration is found in the mcm^5^s^2^U thiolation pathway; sulfur starvation reduces the levels of the Uba4 thiolation enzyme and hence the levels of mcm^5^s^2^U in yeast 13 (Fig. 1). Even if the underlying molecular mechanisms are not fully understood, low mcm^5^s^2^U levels trigger an adaptive response: 1) reduced protein expression due to general slow-down of translation of lysine rich proteins that are found predominantly in the ribosomal machinery; 2) increased levels of methionine, cysteine, and lysine synthesis proteins 13.

The complexity of the responses with the interplay of central regulators such as GCN4 and TOR (Fig. 1), make the dissection of the roles of t^6^A and mcm^5^s^2^U a delicate exercise that will require combining of classical genetic and biochemical studies with the genome wide bioinformatics, proteomic and profiling studies that are now available. These studies are all the more critical as derivatives of both modifications have been linked to human diseases as defects in the ms^2^t^6^A synthesis enzyme have been linked to type 2 diabetes 14, and familial dysautonomia patients have reduced levels of mcm^5^s^2^U 25.
